# Cross-linked Composite Gel Polymer Electrolyte using Mesoporous Methacrylate-Functionalized SiO_2_ Nanoparticles for Lithium-Ion Polymer Batteries

**DOI:** 10.1038/srep26332

**Published:** 2016-05-18

**Authors:** Won-Kyung Shin, Jinhyun Cho, Aravindaraj G. Kannan, Yoon-Sung Lee, Dong-Won Kim

**Affiliations:** 1 Department of Chemical Engineering, Hanyang University, Seoul 04763, South Korea; 2 Center for Energy Convergence, Korea Institute of Science and Technology, Seoul 02792, South Korea

## Abstract

Liquid electrolytes composed of lithium salt in a mixture of organic solvents have been widely used for lithium-ion batteries. However, the high flammability of the organic solvents can lead to thermal runaway and explosions if the system is accidentally subjected to a short circuit or experiences local overheating. In this work, a cross-linked composite gel polymer electrolyte was prepared and applied to lithium-ion polymer cells as a safer and more reliable electrolyte. Mesoporous SiO_2_ nanoparticles containing reactive methacrylate groups as cross-linking sites were synthesized and dispersed into the fibrous polyacrylonitrile membrane. They directly reacted with gel electrolyte precursors containing tri(ethylene glycol) diacrylate, resulting in the formation of a cross-linked composite gel polymer electrolyte with high ionic conductivity and favorable interfacial characteristics. The mesoporous SiO_2_ particles also served as HF scavengers to reduce the HF content in the electrolyte at high temperature. As a result, the cycling performance of the lithium-ion polymer cells with cross-linked composite gel polymer electrolytes employing methacrylate-functionalized mesoporous SiO_2_ nanoparticles was remarkably improved at elevated temperatures.

The rapidly expanding use of rechargeable lithium-ion batteries as power sources for portable electronic devices, electric vehicles and energy storage systems has led to intensive research on electrolyte systems with high electrochemical performance^1^,^2^,^3^,^4^,^5^,^6^,^7^,^8^. The liquid electrolyte used in lithium-ion batteries is based on lithium salt dissolved in a mixture of organic carbonate solvents. It provides high conductivity, acceptable electrochemical stability and good cycle performance. However, current lithium-ion batteries have risks associated with leakage and fire hazards due to the high flammability of the organic solvents^9^,^10^,^11^. In addition, the polyolefin separators used in lithium-ion batteries may shrink and even melt at elevated temperatures, which may cause a short circuit between the two electrodes in cases where unusually high heat is generated^12^,^13^,^14^. Therefore, there is a pressing need for safer and more reliable electrolyte systems. Among various electrolyte systems, gel polymer electrolytes have received considerable attention due to their high ionic conductivity, good interfacial adhesion to electrodes and effective encapsulation of organic solvents in the cell, resulting in suppression of solvent leakage and enhanced safety^15^,^16^,^17^. However, the incorporation of liquid electrolyte into the polymer matrix to improve the ionic conductivity deteriorated the mechanical strength of the polymer electrolyte, leading to internal shorts and battery failure. The mechanical strength of the gel polymer electrolytes was enhanced by *in-situ* chemical cross-linking or addition of inorganic fillers such as SiO_2_, Al_2_O_3_, TiO_2_ and BaTiO_3_^18^,^19^,^20^,^21^,^22^,^23^,^24^,^25^,^26^. In the *in-situ* thermal cross-linking process, gel electrolyte precursors containing liquid electrolyte and cross-linking agents were injected directly into the cell, and a cross-linked gel polymer electrolyte was formed by free radical polymerization triggered by thermal initiation^27^,^28^,^29^. The cross-linked polymer networks that were swelled with the liquid electrolyte showed high ionic conductivity, favorable interfacial properties and good mechanical strength. In our previous studies, we synthesized cross-linked composite polymer electrolytes using reactive SiO_2_ particles with C=C double bonds^30^. The reactive vinyl groups on the surface of the SiO_2_ particles allowed an *in-situ* cross-linking reaction with the gel electrolyte precursor by free radical polymerization. However, these reactive SiO_2_ particles had a non-porous structure, and thus Li^+^ ion transport was blocked by insulating SiO_2_ particles. Therefore, the connectivity of the ion-conducting pathway became more tortuous in these composite gel polymer electrolytes. To solve this problem, we synthesized mesoporous SiO_2_ nanoparticles containing reactive methacrylate groups as inorganic cross-linking sites, and these particles were uniformly dispersed into a fibrous polyacrylonitrile (PAN) membrane. The methacrylate-functionalized SiO_2_ (MA-SiO_2_) nanoparticles in the PAN membrane directly reacted with a gel electrolyte precursor containing tri(ethylene glycol) diacrylate (TEGDA), resulting in the formation of a cross-linked composite gel polymer electrolyte with high ionic conductivity and good interfacial adhesion to the electrodes. The cross-linked composite gel polymer electrolytes synthesized using these mesoporous SiO_2_ particles were utilized in lithium-ion polymer cells with a graphite negative electrode and a LiNi_1/3_Co_1/3_Mn_1/3_O_2_ positive electrode. The cycling performance of the cells was evaluated and compared to those of cells assembled with a cross-linked composite gel polymer electrolyte employing non-porous SiO_2_ nanoparticles.

## Results and Discussion

**Figure 1:**
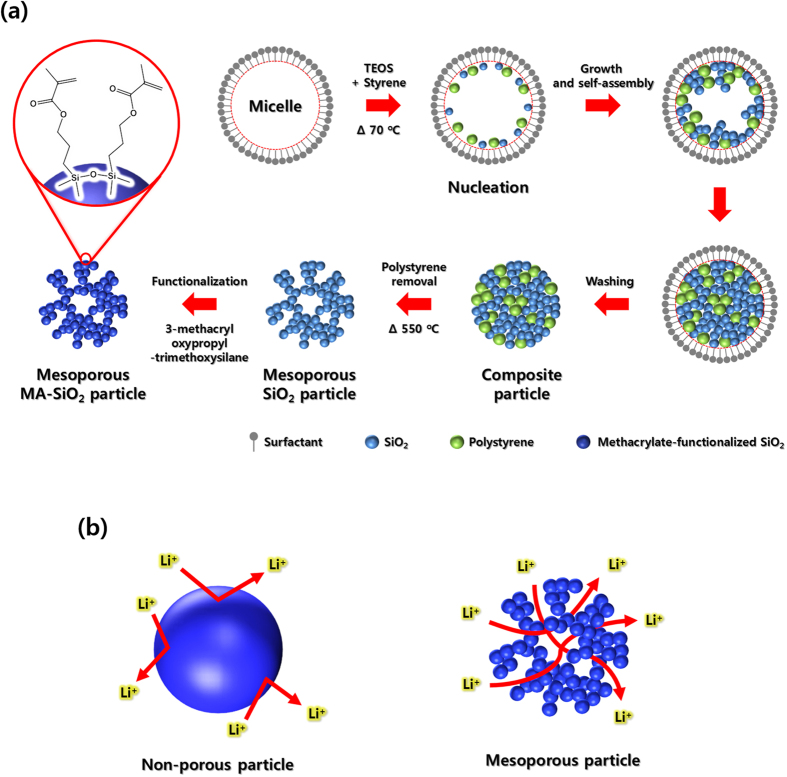
Schematic illustrations for (**a**) synthesis of mesoporous MA-SiO_2_ particles, and (**b**) different lithium ion transport behavior when employing non-porous MA-SiO_2_ particles and mesoporous MA-SiO_2_ particles in the cross-linked composite gel polymer electrolyte.

**Figure 2:**
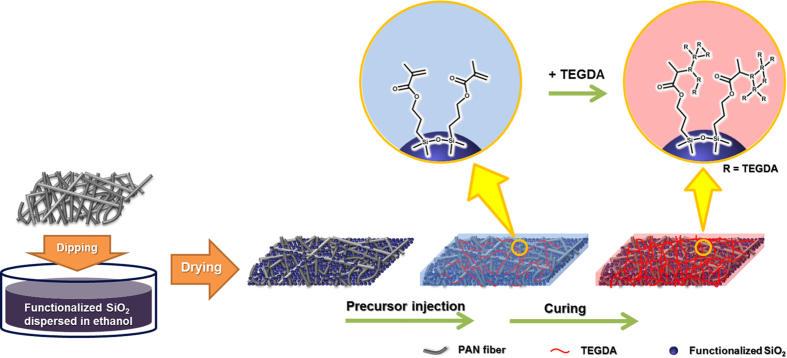
Schematic illustrations of the synthesis of the cross-linked composite gel polymer electrolyte using the fibrous PAN membrane, mesoporous MA-SiO_2_ nanoparticles and gel electrolyte precursor containing TEGDA.

Figure 1a illustrates the scheme for synthesis of mesoporous MA-SiO_2_ nanoparticles^31^. In contrast to the non-porous SiO_2_ particles, lithium ions can pass through mesoporous SiO_2_ particles due to their intra-connected pore network structure, as illustrated in Fig. 1b. A schematic representation of the preparation of cross-linked composite polymer electrolytes using a fibrous PAN membrane and mesoporous MA-SiO_2_ nanoparticles is shown in Fig. 2. The composite membrane with mesoporous MA-SiO_2_ nanoparticles was prepared by simply dipping the fibrous PAN membrane in a solution containing mesoporous MA-SiO_2_ nanoparticles followed by vacuum drying. The composite membrane was used in the lithium-ion cell, and the cell was injected with gel electrolyte precursor containing a small amount of TEGDA and liquid electrolyte. An *in-situ* cross-linking reaction was then initiated at 70 °C for 1 h to form the cross-linked gel polymer electrolyte.

**Figure 3:**
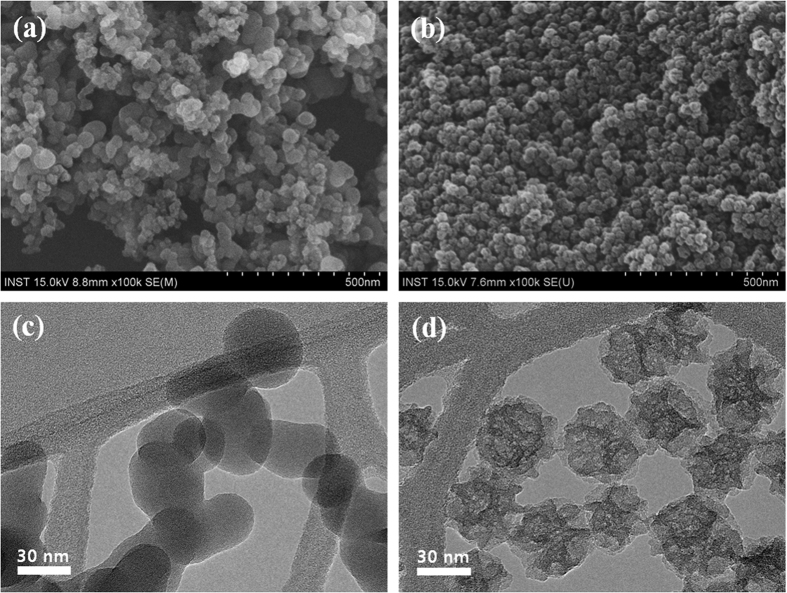
FE-SEM images of (**a**) non-porous MA-SiO_2_ nanoparticles and (**b**) mesoporous MA-SiO_2_ nanoparticles. TEM images of (**c**) non-porous MA-SiO_2_ nanoparticles and (**d**) mesoporous MA-SiO_2_ nanoparticles.

**Figure 4:**
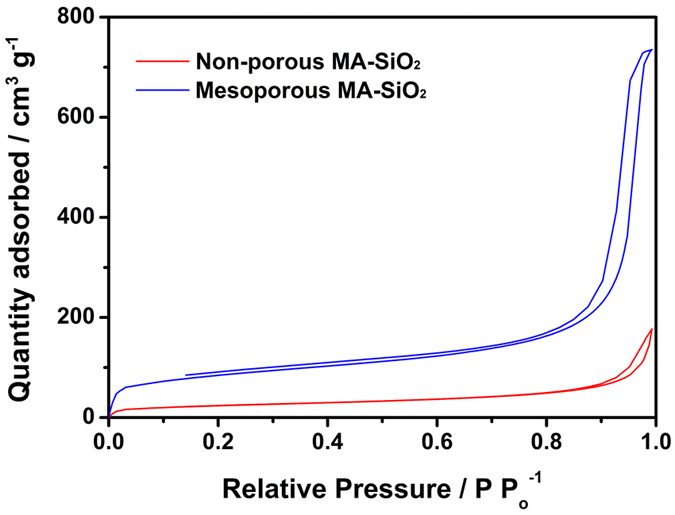
Nitrogen adsorption-desorption isotherms for non-porous MA-SiO_2_ and mesoporous MA-SiO_2_ nanoparticles.

Field emission scanning electron microscope (FE-SEM) images of non-porous and mesoporous MA-SiO_2_ are presented in Fig. 3a,b, respectively. Both particles exhibited spherical shape with an average diameter of around 35 nm. For further comparison, the morphologies of non-porous and mesoporous MA-SiO_2_ nanoparticles were examined using transmission electron microscopy (TEM), as shown in Fig. 3c,d, respectively. We found that the mesoporous MA-SiO_2_ nanoparticles had inner-pore channels that can provide pathways for lithium ions to move through the mesoporous SiO_2_ nanoparticles. In contrast, the non-porous MA-SiO_2_ particles exhibited an impermeable inner structure, indicating that lithium ions cannot pass through the MA-SiO_2_ particles. Figure 4 shows the nitrogen adsorption-desorption isotherms of non-porous and mesoporous MA-SiO_2_ nanoparticles. The isotherm of the mesoporous MA-SiO_2_ nanoparticles exhibited a characteristic Type IV curve. The characteristic feature of the Type IV isotherm is a hysteresis loop, which is associated with capillary condensation taking place in the mesopores; this condensation limits uptake over a range of high relative pressures^32^. Type IV isotherms are typically observed in mesoporous materials^33^,^34^. The Brunauer-Emmet-Teller (BET) surface area of non-porous and mesoporous MA-SiO_2_ nanoparticles were measured to be 86.5 and 292.3 m^2^ g^−1^, respectively. The pore volume of mesoporous MA-SiO_2_ particles was about 1.14 cm^3^ g^−1^, which was much higher than that of non-porous nanoparticles (0.27 cm^3^ g^−1^). These results indicate that the higher surface area of the mesoporous MA-SiO_2_ nanoparticles mainly arose from their higher pore volumes. The pore size distribution of mesoporous MA-SiO_2_ particles is shown in Fig. S1 (Supplementary Information). It shows two different pore sizes with average pore diameters of 9.2 and 15.1 nm, respectively, in which, larger pores (15.1 nm) are predominantly present in the sample. This result confirms that the pores in MA-SiO_2_ particles are mesoporous in nature, which is well consistent with the TEM results.

**Figure 5:**
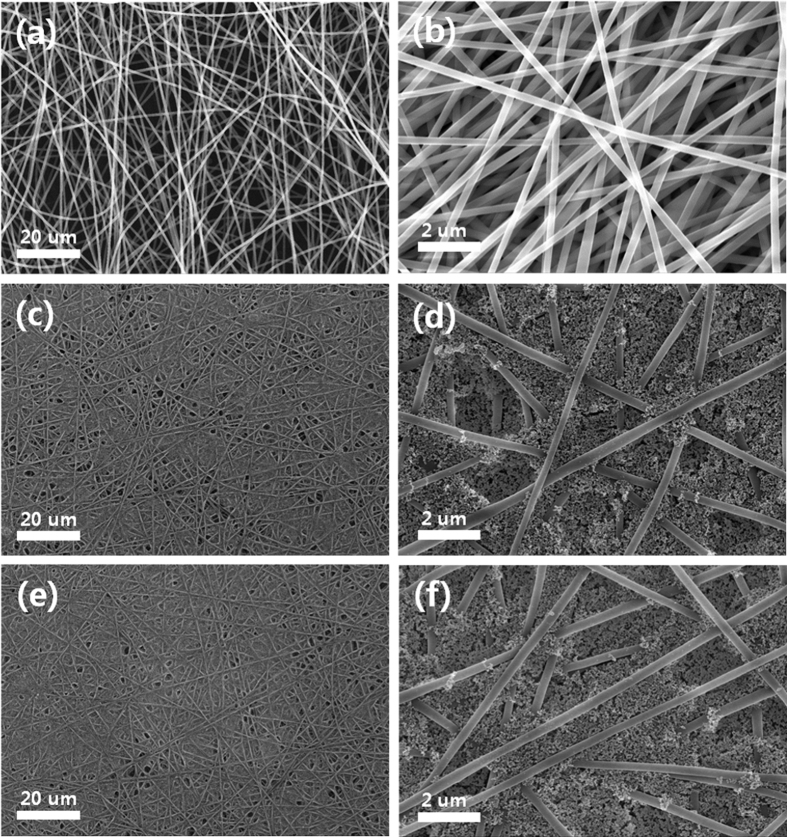
FE-SEM images of electrospun PAN membrane (**a**,**b**), composite PAN membrane with non-porous MA-SiO_2_ nanoparticles (**c,d**) and composite PAN membrane with mesoporous MA-SiO_2_ nanoparticles (**e,f**) at two different magnifications.

Figure 5a,b present FE-SEM images of an electrospun PAN membrane at two different magnifications, which show a highly porous and interconnected three-dimensional fibrous network structure with micron-sized pores. The fibrous PAN membranes were dipped in a solution containing MA-SiO_2_ nanoparticles, and the membranes were then dried. As a result, both non-porous and mesoporous MA-SiO_2_ nanoparticles were uniformly close-packed in the large pores of the fibrous PAN membrane, as shown in Fig. 5c–f. These MA-SiO_2_ nanoparticles have reactive C=C double bonds on their surface, and thus they can serve as cross-linking sites through free radical polymerization in the fibrous PAN membrane. The thicknesses of the composite membranes containing non-porous and mesoporous MA-SiO_2_ nanoparticles were measured to be 40 μm, which was about the same as the fibrous PAN membranes without MA-SiO_2_ nanoparticles. This result suggests that most of the MA-SiO_2_ nanoparticles were embedded into the large pores of the fibrous PAN membrane rather than merely residing on the surface. A thermal cross-linking reaction was carried out using the composite membrane, gel electrolyte precursor containing TEGDA and liquid electrolyte to form a cross-linked composite gel polymer electrolyte. Before cell assembly, FT-IR analysis was performed to confirm the chemical cross-linking reaction between mesoporous MA-SiO_2_ nanoparticles and TEGDA, and the resulting FT-IR spectra are shown in Fig. S2. The pristine fibrous PAN membrane showed peaks at 2240 and 1446 cm^−1^, which corresponded to C-N stretching vibrations and C-H bending vibrations, respectively^35^. When the MA-SiO_2_ nanoparticles were added into the fibrous PAN membrane, peaks corresponding to the siloxane (Si-O-Si) group (1190 and 1082 cm^−1^), C=O bonds (1721 cm^−1^) and C=C double bonds (1636 cm^−1^) were additionally observed^36^,^37^,^38^, indicating that MA-SiO_2_ nanoparticles containing methacrylate groups were well incorporated in the porous PAN membrane. The FT-IR spectrum of the cross-linked composite gel polymer electrolyte revealed that the peaks corresponding to C=C double bonds in the MA-SiO_2_ particles (1636 cm^−1^) and TEGDA (1618 cm^−1^) disappeared after thermal cross-linking. This result indicates that methacrylate groups on the surface of MA-SiO_2_ particles reacted with TEGDA through free radical polymerization to form cross-linked gel polymer electrolytes. The electrolyte solution was well encapsulated in the cross-linked composite gel polymer electrolyte, and the organic solvents did not leak out. Also, the adhesion between the PAN membrane and the SiO_2_ nanoparticles is very strong, since the mesoporous MA-SiO_2_ particles contain many reactive groups that participate in radical polymerization with TEGDA during cross-linking reaction, which prevents SiO_2_ particles from detaching from the PAN membrane. The mechanical strength of the cross-linked PAN-based polymer membranes was measured, and the results are shown in Fig. S3. It is clearly seen that the incorporation of mesoporous MA-SiO_2_ particles into the PAN membrane improved the tensile strength. This result implies that the mesoporous and reactive SiO_2_ nanoparticles with high mechanical strength participate in the chemical cross-linking reaction with TEGDA as cross-linking sites, thereby resulting in increase of degree of cross-linking and mechanical strength. To investigate the distribution of MA-SiO_2_ nanoparticles in the fibrous PAN membrane after the thermal cross-linking reaction, energy dispersive X-ray spectroscopy (EDS) mapping images of nitrogen and silicon elements in the cross-linked composite gel polymer electrolyte were obtained, as shown in Fig. S4. The silicon arising from MA-SiO_2_ nanoparticles was evenly distributed in the cross-linked composite gel polymer electrolyte, indicating homogeneous dispersion of MA-SiO_2_ nanoparticles.

**Figure 6:**
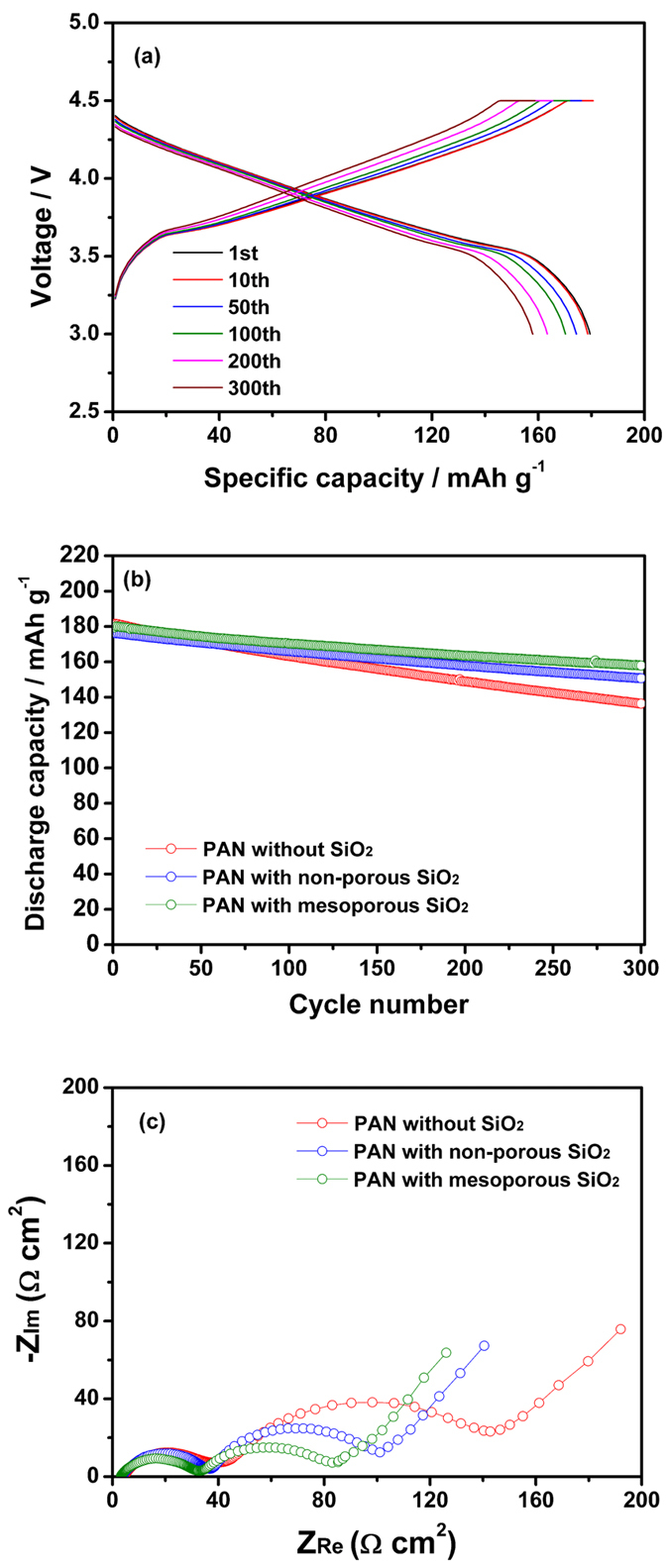
(**a**) Charge and discharge curves of lithium-ion polymer cell assembled with the cross-linked composite gel polymer electrolyte using mesoporous MA-SiO_2_ nanoparticles and (**b**) discharge capacities of lithium-ion polymer cells assembled with different electrolytes at 25 °C (0.5 C CC & CV charge, 0.5 C CC discharge, cut-off voltage: 3.0–4.5 V). (**c**) AC impedance spectra of lithium-ion polymer cells assembled with different electrolytes, which were measured after 300 cycles.

We evaluated the cycling performance of lithium-ion polymer cells assembled using an *in-situ* cross-linking reaction with the composite membranes containing MA-SiO_2_ nanoparticles and gel electrolyte precursor. The cell was initially subjected to a preconditioning cycle over a voltage range of 3.0–4.5 V at a rate of 0.1 C. After two cycles at the 0.1 C rate, the cell was cycled in the same voltage range at a 0.5 C rate (0.8 mA cm^−2^). Figure 6a shows the voltage profiles of the lithium-ion polymer cell assembled with a cross-linked composite gel polymer electrolyte employing mesoporous MA-SiO_2_ particles. The cell initially delivered a discharge capacity of 179.5 mAh g^−1^ based on active LiNi_1/3_Co_1/3_Mn_1/3_O_2_ material in the positive electrode. It delivered a discharge capacity of 157.9 mAh g^−1^ after 300 cycles, corresponding to 88.0% of the initial discharge capacity. Figure 6b compares the discharge capacities of the cells prepared with composite membranes containing different types of MA-SiO_2_ particles (non-porous MA-SiO_2_ particles and mesoporous MA-SiO_2_ particles) as a function of cycle number. For the purpose of comparison, the cycling results of the cell assembled with cross-linked gel polymer electrolyte using a fibrous PAN membrane (without MA-SiO_2_ particles) and gel electrolyte precursor are also shown. The initial discharge capacity of the cell was slightly decreased when using cross-linked composite gel polymer electrolytes with MA-SiO_2_ particles. The presence of reactive MA-SiO_2_ particles in the composite membrane may increase the degree of cross-linking and increase the resistance of ion migration in the cell, resulting in a reduction in discharge capacity. The cell assembled with mesoporous MA-SiO_2_ particles delivered higher discharge capacity than the cell with non-porous MA-SiO_2_ particles. This result can be ascribed to the higher ionic conductivity of the cross-linked composite gel polymer electrolyte employing mesoporous MA-SiO_2_ particles. The ionic conductivities of the cross-linked composite gel polymer electrolytes prepared with non-porous MA-SiO_2_ particles and mesoporous MA-SiO_2_ particles were 1.1 × 10^−3^ and 1.8 × 10^−3^ S cm^−1^, respectively. As mentioned earlier, the mesoporous SiO_2_ nanoparticles had many pores that provided an ion-conduction pathway through mesoporous SiO_2_ particles, resulting in a higher ionic conductivity than the cross-linked composite gel polymer electrolyte with non-porous SiO_2_ particles. With respect to capacity retention, the cell assembled with mesoporous MA-SiO_2_ particles exhibited the best cycling stability among the cells investigated. The presence of mesoporous MA-SiO_2_ particles with large surface areas in the fibrous PAN membrane allowed for increased cross-linking, which resulted in strong interfacial contacts with electrodes. Moreover, the effective encapsulation of liquid electrolyte in the cross-linked composite gel polymer electrolyte prevented leakage of the electrolyte solution during cycling, resulting in good cycling stability. To examine the effect of mesoporous MA-SiO_2_ particles on the cycling performance of the cells, AC impedance measurements were performed for each type of cell after 300 cycles, and the resulting AC impedance spectra are shown in Fig. 6c. All spectra exhibited two overlapping semicircles due to different interfacial resistance contributions. The first semicircle in the higher frequency range was attributed to resistance due to Li^+^ ion migration through the surface film on the electrodes (R_f_), and the second semicircle in the middle to low frequency range arises from charge transfer resistance at the electrode-electrolyte interface (R_ct_)^39^,^40^. The electrolyte resistance estimated from the intercept on the real axis in the high-frequency range was the lowest in the cell employing mesoporous MA-SiO_2_ particles. In addition, the cell with mesoporous SiO_2_ particles had the lowest interfacial resistance after 300 cycles. In this cell, the cross-linked composite gel polymer electrolyte provided effective encapsulation of the electrolyte solution, and thus the deleterious reactions between the electrodes and the electrolyte were reduced, which resulted in limited growth of the resistive layer on electrode surface and enhanced interfacial stability. *In-situ* cross-linking in the presence of mesoporous MA-SiO_2_ particles with large surface areas also helped to intimately adhere the PAN membrane to the electrodes, thereby providing favorable charge transport between the electrolyte and electrodes. These results suggest that *in-situ* cross-linking of an electrolyte solution in the presence of mesoporous MA-SiO_2_ particles rather than non-porous MA-SiO_2_ particles was more effective for maintaining good interfacial contact between the electrodes and the electrolyte, and also improved retention of the electrolyte solution in the cell during cycling.

**Figure 7:**
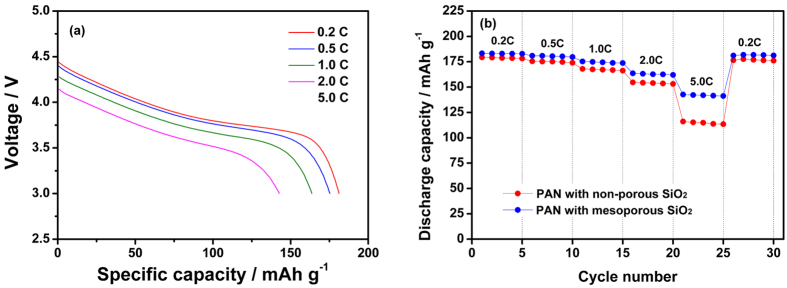
(**a**) Discharge curves of lithium-ion polymer cell assembled with cross-linked composite gel polymer electrolyte employing mesoporous MA-SiO_2_ particles and (**b**) discharge capacities of lithium-ion polymer cells assembled with different electrolytes as a function of C rate.

The rate capability of the lithium-ion polymer cell assembled with different electrolytes was evaluated at various C rates. Voltage profiles of the cell prepared with cross-linked composite gel polymer electrolyte employing mesoporous MA-SiO_2_ particles are presented in Fig. 7a. Although the overpotential gradually increased with increasing current rate, the cell delivered a high discharge capacity of 142.7 mAh g^−1^ at a 5.0 C rate. Figure 7b compares the discharge capacities of the lithium-ion polymer cells assembled with non-porous MA-SiO_2_ particles and mesoporous MA-SiO_2_ particles at different current rates. In this test, the C rate gradually increased from 0.2 to 5.0 C every five cycles. Comparison of the rate capabilities of the two cells showed that the use of mesoporous MA-SiO_2_ particles improved the high rate performance. The cell with mesoporous MA-SiO_2_ particles also exhibited an ability to recover its capacity when the C rate decreased from 5.0 to 0.2 C. These results demonstrate that the use of mesoporous MA-SiO_2_ particles not only enhanced the rate capability, but also increased the cycling stability of the cell.

**Figure 8:**
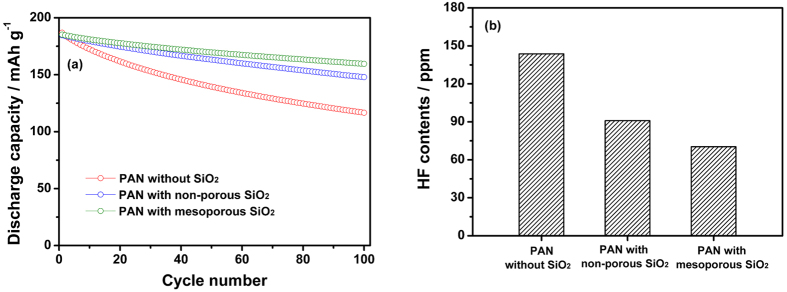
(**a**) Discharge capacities of lithium-ion polymer cells assembled with different electrolytes at 55 °C (0.5 C CC & CV charge, 0.5 C CC discharge, cut-off voltage: 3.0–4.5 V). (**b**) HF content in the different electrolytes after being stored at 55 °C for 3 days.

Figure 8a shows the discharge capacities the lithium-ion polymer cells assembled with different electrolytes as a function of cycle number, which are obtained at 55 °C and 0.5 C rate. The cells delivered initial discharge capacities ranging from 184.7 to 187.0 mAh g^−1^, which were slightly higher than those obtained at 25 °C. The capacity retention at elevated temperature was greatly improved by using the cross-linked composite gel polymer electrolyte with mesoporous MA-SiO_2_ particles. The capacity fading of layered LiNi_x_Co_y_Mn_1-x-y_O_2_ electrodes at high temperatures is mainly due to structural instabilities as well as dissolution of transition metals from the active cathode material by HF attack^41^,^42^. HF is known to be generated by thermal decomposition and hydrolysis of LiPF_6_ by trace moisture in the electrolyte solution. Figure 8b shows the HF concentrations in the cells with different electrolytes; these values were measured after storing the cells at 55 °C for three days. Clearly, the HF content was significantly reduced in the cells with mesoporous MA-SiO_2_ particles. When mesoporous MA-SiO_2_ particles are exposed to the electrolyte solution containing HF, protonation of oxygen occurs by Bronsted acid–Lewis base reaction. Since the resulting oxonium intermediate is very unstable, the fluoride anions readily attack Si to form strong chemical bonds (Si–F) by a nucleophilic substitution reaction^43^. Since the nucleophilic substitution reaction of HF mainly occurred on the surface of the SiO_2_ particles, the mesoporous MA-SiO_2_ nanoparticles with high surface area were more effective in reducing HF concentration. Accordingly, the use of mesoporous MA-SiO_2_ particles with large surface area reduced HF content and suppressed the dissolution of transition metals from the active LiNi_1/3_Co_1/3_Mn_1/3_O_2_ material at elevated temperatures, thereby exhibiting stable cycling behavior at elevated temperatures.

## Conclusion

Mesoporous methacrylate-functionalized SiO_2_ nanoparticles were synthesized and embedded into a fibrous PAN membrane. A cross-linked composite gel polymer electrolyte was then prepared by an *in-situ* cross-linking reaction using the composite membrane employing mesoporous MA-SiO_2_ nanoparticles and electrolyte solutions containing a small amount of TEGDA. The cross-linked composite gel polymer electrolyte effectively encapsulated the electrolyte solution without solvent leakage and exhibited favorable interfacial characteristics. The *in-situ* chemical cross-linking using the mesoporous SiO_2_ nanoparticles was more effective than non-porous SiO_2_ nanoparticles for obtaining good cycling performance in terms of discharge capacity, capacity retention, rate capability and high temperature cycling stability.

## Methods

### Synthesis of mesoporous MA-SiO_2_ nanoparticles

Mesoporous silica nanoparticles were synthesized as reported in earlier literature^31^. Briefly, 0.4 g of cetyltrimethylammonium bromide (CTAB, Daejung) and 0.045 g of L-lysine (TCI Co. Ltd.) were added to 140 ml of a mixture of water/octane (10:1 by volume), and this solution was stirred at 70 °C in a three-necked flask reactor. After a clear solution was obtained, 3.3 g of styrene (Kanto Chemical Co.), 4.0 g of tetraorthosilicate (TEOS, Evonik) and 0.14 g of azobis(2-methylpropionamide) dihydrochloride (AIBA, Sigma-Aldrich) were added, and the mixture was stirred for 20 h at 70 °C under a nitrogen atmosphere. After the reaction, the resulting solution was centrifuged and washed with ethanol several times. The polystyrene template was completely removed by heating at 550 °C for 10 h to obtain mesoporous SiO_2_ nanoparticles. Surface modification of mesoporous SiO_2_ nanoparticles was carried out by using 3-methacryloxypropyltrimethoxysilane (MEMO, Evonik)^44^. 0.5 g of silica nanoparticles was dispersed in methanol via ultrasonication for 30 min, and 10.3 mmol L^−1^ of MEMO was added into the solution. The mixture was stirred for 1 h in order to induce surface functionalization with methacrylate groups. The resulting solution was centrifuged and washed with methanol several times. Mesoporous MA-SiO_2_ nanoparticles were finally obtained as a white powder after vacuum drying at 70 °C for 12 h.

### Preparation of fibrous PAN membrane with mesoporous MA-SiO_2_ nanoparticles

The electrospun fibrous PAN membrane was prepared using an electrospinning method, as previously reported^45^. PAN (M_w_ = 150,000, Sigma-Aldrich) was dissolved in anhydrous N,N-dimethylformamide at 60 °C to a concentration of 10 wt.%, and the resulting polymer solution was injected through a capillary tip using a plastic syringe (10 ml). During electrospinning, a high voltage of 11 kV was applied to the needle, and the flow rate of the spinning solution was controlled to 0.8 ml h^−1^. The distance between the tip and the rotating drum collector was 16 cm, and the metal drum was rotated at 200 rpm. The electrospun PAN fibers were collected on an aluminum foil wrapped around the drum, and they were dried overnight in a vacuum oven at 100 °C. The thickness of the PAN membrane was controlled to be about 40 μm. The coating solution was prepared by dispersing 5 wt.% of the MA-SiO_2_ nanoparticles in ethanol by sonication for 1 h. The electrospun PAN membrane was then soaked in the coating solution, and the resulting composite PAN membrane was dried at 70 °C for 24 h.

### Electrode preparation and cell assembly

The positive electrode was prepared by coating an N-methyl pyrrolidone (NMP)-based slurry containing LiNi_1/3_Co_1/3_Mn_1/3_O_2_ (3M Co.), poly(vinylidene fluoride) (PVdF) and super-P carbon (MMM Co.) (85:7.5:7.5 by weight) onto aluminum foil. The electrode was dried under vacuum at 110 °C for 12 h. The electrode dimension was 2.8 × 3.8 cm^2^, and its active material loading corresponded to a capacity of about 1.6 mAh cm^−2^. The negative electrode was prepared similarly by coating the NMP-based slurry of mesocarbon microbeads (MCMB, Osaka gas), PVdF and super-P carbon (85/7.5/7.5 by weight) onto a copper foil. To prepare gel electrolyte precursor, 4 wt.% of TEGDA (Sigma-Aldrich) was added to liquid electrolyte with azobis(isobutyronitrile) (Junsei, 1 wt.% of TEGDA) as a thermal radical initiator. 1.15 M LiPF_6_ in ethylene carbonate (EC)/ethylmethyl carbonate (EMC) (3:7 by volume, PANAX ETEC Co. Ltd., battery grade) was used as the liquid electrolyte. The lithium-ion polymer cell was assembled by sandwiching a composite membrane between a graphite negative electrode and a LiNi_1/3_Co_1/3_Mn_1/3_O_2_ positive electrode. The cell was enclosed in a pouch injected with gel electrolyte precursor and was then vacuum-sealed. After the cell assembly, the cells were stored at 70 °C for 1 h in order to induce the *in-situ* chemical cross-linking reaction between the gel electrolyte precursor and the mesoporous MA-SiO_2_ nanoparticles. All cells were assembled in a dry box filled with argon gas.

### Characterization and measurements

The morphologies of the mesoporous MA-SiO_2_ nanoparticles and fibrous PAN membrane were examined using FE-SEM (JEOL JSM-6330F) and TEM (JEOL JEM-2010). EDS mapping was used for morphological assessment and elemental characterization in the cross-linked composite gel polymer electrolyte. BET surface areas were measured by a nitrogen adsorption-desorption method using a 3 Flex Surface Characterization Analyzer (Micromeritics) and the pore size distribution was obtained from the analysis of adsorption branch of the isotherm using Barret-Joyner-Halenda (BJH) method. FT-IR spectra were obtained using a Magna IR 760 spectrometer in the range of 400–4,000 cm^−1^ with KBr powder-pressed pellets. The mechanical properties of the cross-linked polymer membranes were measured using a universal test machine (Instron 5966) in accordance with the ASTM D882 method. Charge and discharge cycling tests of the lithium-ion polymer cells were conducted at a current density of 0.8 mA cm^−2^ (0.5 C rate) over a voltage range of 3.0–4.5 V using a battery cycler (WBCS 3000, WonA Tech Co., Ltd.) at 25 and 55 °C, respectively. AC impedance measurements were performed using an impedance analyzer (IM6, Zahner Electrik) over the frequency range of 5 mHz to 100 kHz with an amplitude of 10 mV. HF content in the electrolyte was measured using an acid-base titration method after the cell was stored in a 55 °C oven for 3 days. Methyl orange (Sigma-Aldrich) was used as an acid–base indicator.

## Additional Information

**How to cite this article**: Shin, W.-K. *et al.* Cross-linked Composite Gel Polymer Electrolyte using Mesoporous Methacrylate-Functionalized SiO_2_ Nanoparticles for Lithium-Ion Polymer Batteries. *Sci. Rep.* **6**, 26332; doi: 10.1038/srep26332 (2016).

## Supplementary Material

Supplementary Information
