# Supplementary material for: Cross-linked Composite Gel Polymer Electrolyte using Mesoporous Methacrylate-Functionalized SiO2 Nanoparticles for Lithium-Ion Polymer Batteries
Source: Sci Rep. 2016 May 18;6:26332. doi: 10.1038/srep26332 (PMC4870681; doi:10.1038/srep26332)
Supplement: Supplementary Information [file srep26332-s1.pdf]

Supplementary Information

Cross-linked Composite Gel Polymer Electrolyte using Mesoporous Methacrylate-Functionalized SiO_2 Nanoparticles for Lithium-Ion Polymer Batteries

Won-Kyung Shin[1], Jinhyun Cho[1], Aravindaraj G. Kannan[1], Yoon-Sung Lee[2] & Dong-Won Kim[1,*]

[1]Department of Chemical Engineering, Hanyang University, Seoul 04763, South Korea

[2]Center for Energy Convergence, Korea Institute of Science and Technology, Seoul 02792, South Korea

*dongwonkim@hanyang.ac.kr

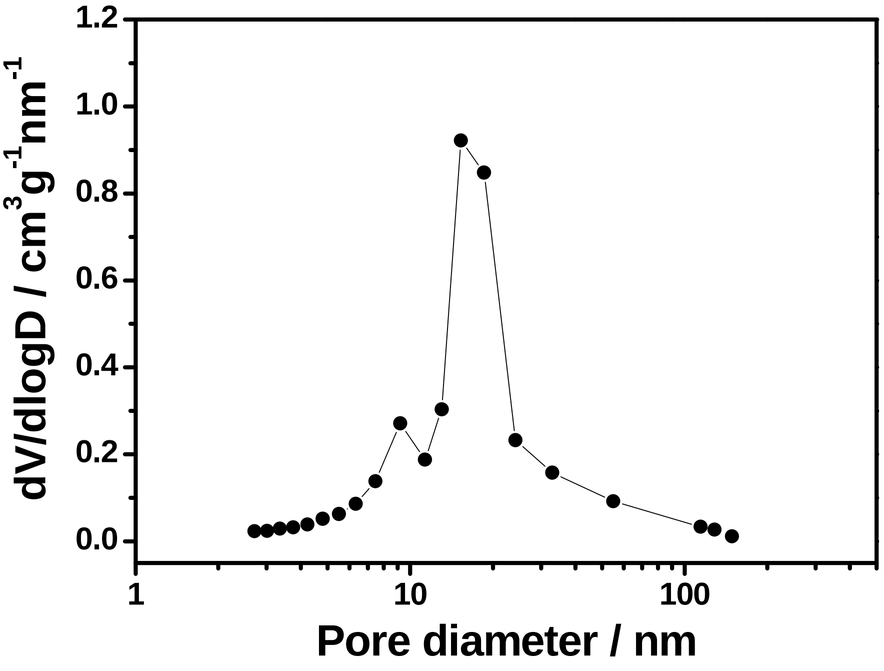

Figure S1. Pore size distribution of the mesoporous MA-SiO_2 particles.

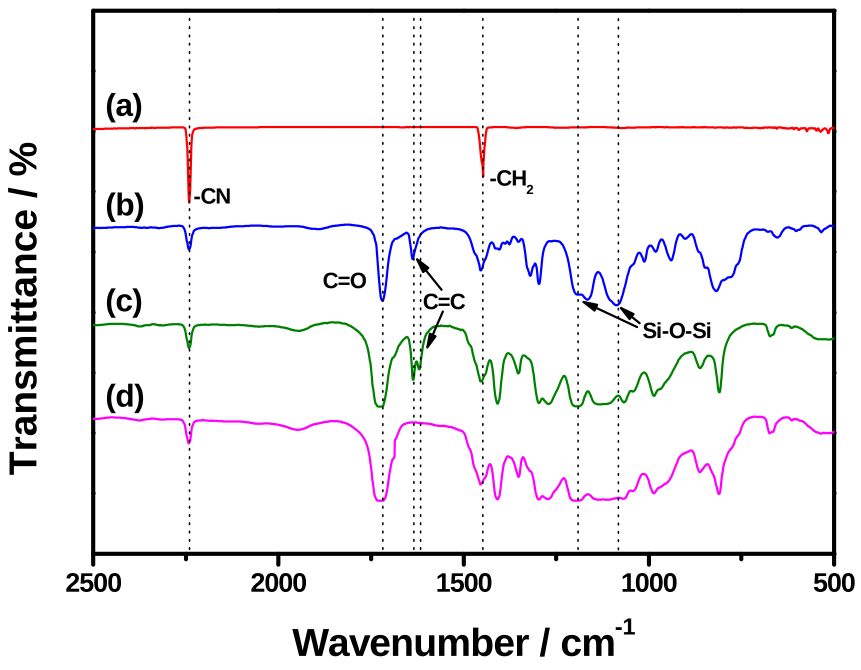

Figure S2. FT-IR spectra of (a) fibrous PAN membrane, (b) composite PAN membrane with mesoporous MA-SiO_2 nanoparticles, and cross-linked composite polymer electrolyte (c) before and (d) after thermal cross-linking.

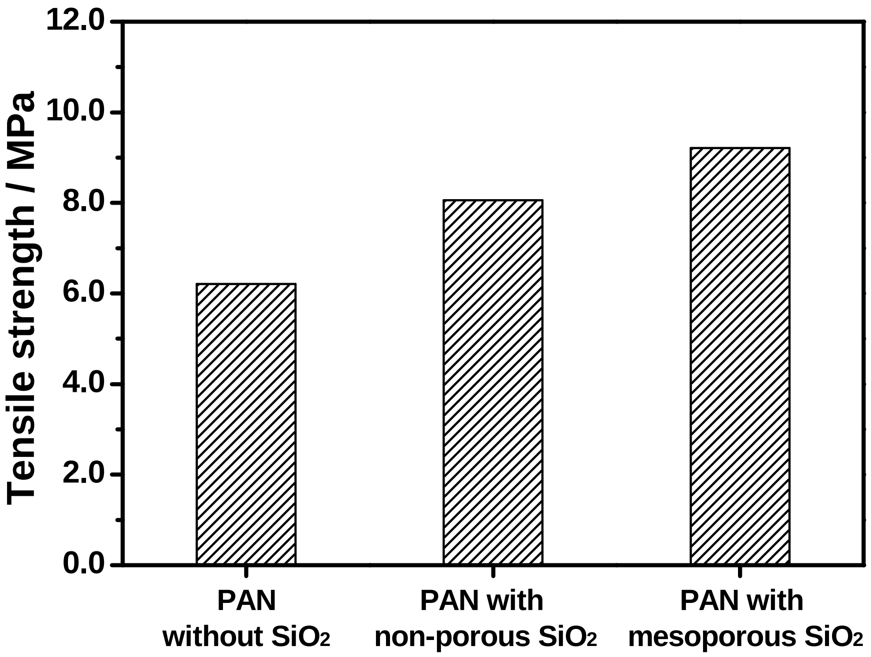

Figure S3. Mechanical strength of cross-linked PAN-based polymer membranes containing different types of SiO_2 particles.

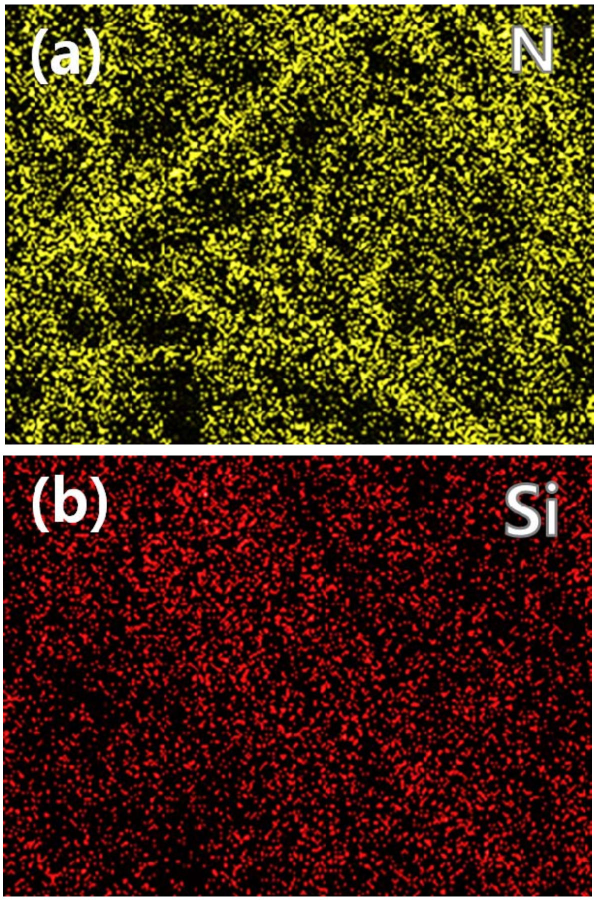

Figure S4. EDS elemental mapping images corresponding to (a) N and (b) Si in the cross-linked composite gel polymer electrolyte with mesoporous MA-SiO_2 nanoparticles.
